# Signal Detection of Pediatric Drug–Induced Coagulopathy Using Routine Electronic Health Records

**DOI:** 10.3389/fphar.2022.935627

**Published:** 2022-07-20

**Authors:** Xiaolu Nie, Yuncui Yu, Lulu Jia, Houyu Zhao, Zhenping Chen, Liqiang Zhang, Xiaoling Cheng, Yaqi Lyu, Wang Cao, Xiaoling Wang, Xiaoxia Peng

**Affiliations:** ^1^ Center for Clinical Epidemiology and Evidence-based Medicine, National Center for Children’s Health, Beijing Children’s Hospital, Capital Medical University, Beijing, China; ^2^ Department of Epidemiology and Biostatistics, School of Public Health, Peking University, Beijing, China; ^3^ Hainan Institute of Real World Data, Qionghai, China; ^4^ Department of Pharmacy, Beijing Children’s Hospital, Capital Medical University, National Center for Children’s Health, Beijing, China; ^5^ Hematologic Disease Laboratory, National Center for Children’s Health, Beijing Pediatric Research Institute, Beijing Children’s Hospital, Capital Medical University, Beijing, China; ^6^ Hematology Center, National Center for Children’s Health, Beijing Children’s Hospital, Capital Medical University, Beijing, China; ^7^ Department of Medical Record Management, Beijing Children’s Hospital, Capital Medical University, National Center for Children’s Health, Beijing, China

**Keywords:** drug-induced coagulopathy, children, signal detection, electronic health records, post-marketing pharmacovigilance, drug scientific supervision

## Abstract

**Background:** Drug-induced coagulopathy (DIC) is a severe adverse reaction and has become a significantly increased clinical problem in children. It is crucial to the detection of the DIC safety signal for drug post-marketing scientific supervision purposes. Therefore, this study aimed to detect potential signals for DIC in children using the routine electronic medical record (EMR) data.

**Methods:** This study extracted EMR data from Beijing Children’s Hospital between 2009 and 2020. A two-stage modeling method was developed to detect the signal of DIC. We calculated the crude incidence by mining cases of coagulopathy to select the potential suspected drugs; then, propensity score-matched retrospective cohorts of specific screened drugs from the first stage were constructed and estimated the odds ratio (OR) and 95% confidence interval (CI) using conditional logistic regression models. The current literature evidence was used to assess the novelty of the signal.

**Results:**In the study, from a total of 340 drugs, 22 drugs were initially screened as potentially inducing coagulopathy. In total, we identified 19 positive DIC associations. Of these, potential DIC risk of omeprazole (OR: 2.23, 95% CI: 1.88–2.65), chlorpheniramine (OR:3.04, 95% CI:2.56–3.60), and salbutamol sulfate (OR:1.36, 95% CI:1.07–1.73) were three new DIC signals in both children and adults. Twelve associations between coagulopathy and drugs, meropenem (OR: 3.38, 95% CI: 2.72–4.20), cefoperazone sulbactam (OR: 2.80, 95% CI: 2.30–3.41), fluconazole (OR: 2.11, 95% CI: 1.71–2.59), voriconazole (OR: 2.82, 95% CI: 2.20–3.61), ambroxol hydrochloride (OR: 2.12, 95% CI: 1.74–2.58), furosemide (OR: 2.36, 95% CI: 2.08–2.67), iodixanol (OR: 2.21, 95% CI: 1.72–2.85), cefamandole (OR: 1.82, 95% CI: 1.56–2.13), ceftizoxime (OR: 1.95, 95% CI: 1.44–2.63), ceftriaxone (OR: 1.95, 95% CI: 1.44–2.63), latamoxef sodium (OR: 1.76, 95% CI: 1.49–2.07), and sulfamethoxazole (OR: 1.29, 95% CI: 1.01–1.64), were considered as new signals in children.

**Conclusion:** The two-stage algorithm developed in our study to detect safety signals of DIC found nineteen signals of DIC, including twelve new signals in a pediatric population. However, these safety signals of DIC need to be confirmed by further studies based on population study and mechanism research.

## 1 Introduction

Drug-induced coagulopathy (DIC) is an adverse drug reaction (ADR) that manifests as derangement of hemostasis and is a significantly under-recognized clinical problem, especially in surgical patients (Peralta et al., 2019); 7.2 out of 100 patients taking anticoagulants require management for DIC (Nalezinski, 2022). The prothrombin time (PT) or the activated partial thromboplastin time (APTT) is usually longer than the upper limits of normal (Hiensch and Lee, 2021); therefore, DIC can often lead to abrupt and excessive bleeding complications and even death (Hiensch and Lee, 2021). Early identification and timely correction of DIC in surgical patients or emergency department patients with significant bleeding are paramount to prevent death and other consequences of hemorrhage (Liu et al., 2019). It has been reported that many kinds of medications, including vitamin K antagonists, direct oral anticoagulants, and antibiotics, could lead to DIC in the adult population (Zengotita and Holt, 1986; Hall and Carson, 2012; Liu et al., 2019). However, the current pediatric drug safety landscape, including clinical trials, is limited as it rarely includes children and relies on extrapolation from adults. Children have immature organ function and a different spectrum of diseases compared with adults, and it is proposed that pediatric drug safety should comprehensively consider children’s systems biology (Giangreco et al., 2022). Hence, accurate methods for post-marketing drug safety surveillance and signal detection of DIC in children are urgently needed.

Considering the limitation of the passive surveillance system using spontaneous reporting system databases such as the FDA Adverse Event Reporting System (FAERS) and EudraVigilance, ADR active surveillance using real-world data (RWD), electronic medical records (EMR) for instance, has opened a new era in pharmacovigilance (Lee et al., 2020). The depth and breadth of clinical data within EHR systems paired with innovative data-mining methods can be leveraged to detect novel drug safety signal, especially the off-label drug use that often occur in pediatric patients (Rudin et al., 2020). Several studies have been conducted to develop methods for detecting signals of hematological disorders using RWD databases (Fuzier et al., 2013; Nie et al., 2021). However, these studies mainly focused on adult patients, and, to date, little is known about children.

This study aimed to develop a two-stage procedure to detect signals of DIC in the child population using EMR data and provide candidate drugs for further precise drug monitoring and precision medicine in pediatrics.

## 2 Method

The study was conducted in accordance with the Declaration of Helsinki. The protocol was approved by the Institutional Review Board (IRB) of Beijing Children’s Hospital, Capital Medical University (approval number: 2018-129), with a waiver of informed consent. All the data we used have been de-identified to protect patient privacy and confidentiality. This study was reported critically according to the RECORD-PE statement.

### 2.1 Data Sources

This retrospective cohort study was conducted using data on hospitalized patients in Beijing Children’s Hospital (BCH) longitudinal inpatient database from 1 January 2009 to 31 December 2020, which has been described previously (Nie et al., 2021). These data encompassed health information including medical orders of doctors, diagnosis records from hospital information systems, laboratory tests from laboratory information systems, and drug prescriptions. If a person with the same patient ID had multiple hospital admissions, we identified these records as different records. Therefore, there were approximately 5,75,965 records of inpatients under 18 years of age.

### 2.2 Study Population Identification

Eligible participants were hospitalized patients aged 28 days to 18 years old and had at least two times laboratory test records of any of the two kinds of main coagulation function index for PT or APTT as well as drug prescriptions in the data warehouse. Considering the temporal relationship between suspected drug and coagulopathy events is important for safety signal identification, patients whose initial PT or APTT index at the beginning of the study was out of reference interval (i.e., PT > 12.5 s or PT < 9.4 s APTT > 38.4 s or APTT < 25.1 s) were excluded.

### 2.3 Laboratory Criterion of DIC

The laboratories of BCH are certified and accredited under the appropriate International Organization for Standardization standards. According to the definition of coagulopathy in the published Mount Sinai Expert Guides: Critical Care in 2021 (3), the reference interval of pediatric coagulation parameters (Jiang et al., 2015), and the method of the IHI Global ADR Trigger Tool (Griffin and Resar, 2009), the trigger of pediatric DIC in this study was defined as PT longer than 12.5 s or APTT longer than 38.4 s after administration of a particular medicine within the appropriate therapeutic dose range.

### 2.4 Development of a Two-Stage Signal Detection Model

**FIGURE 1:**
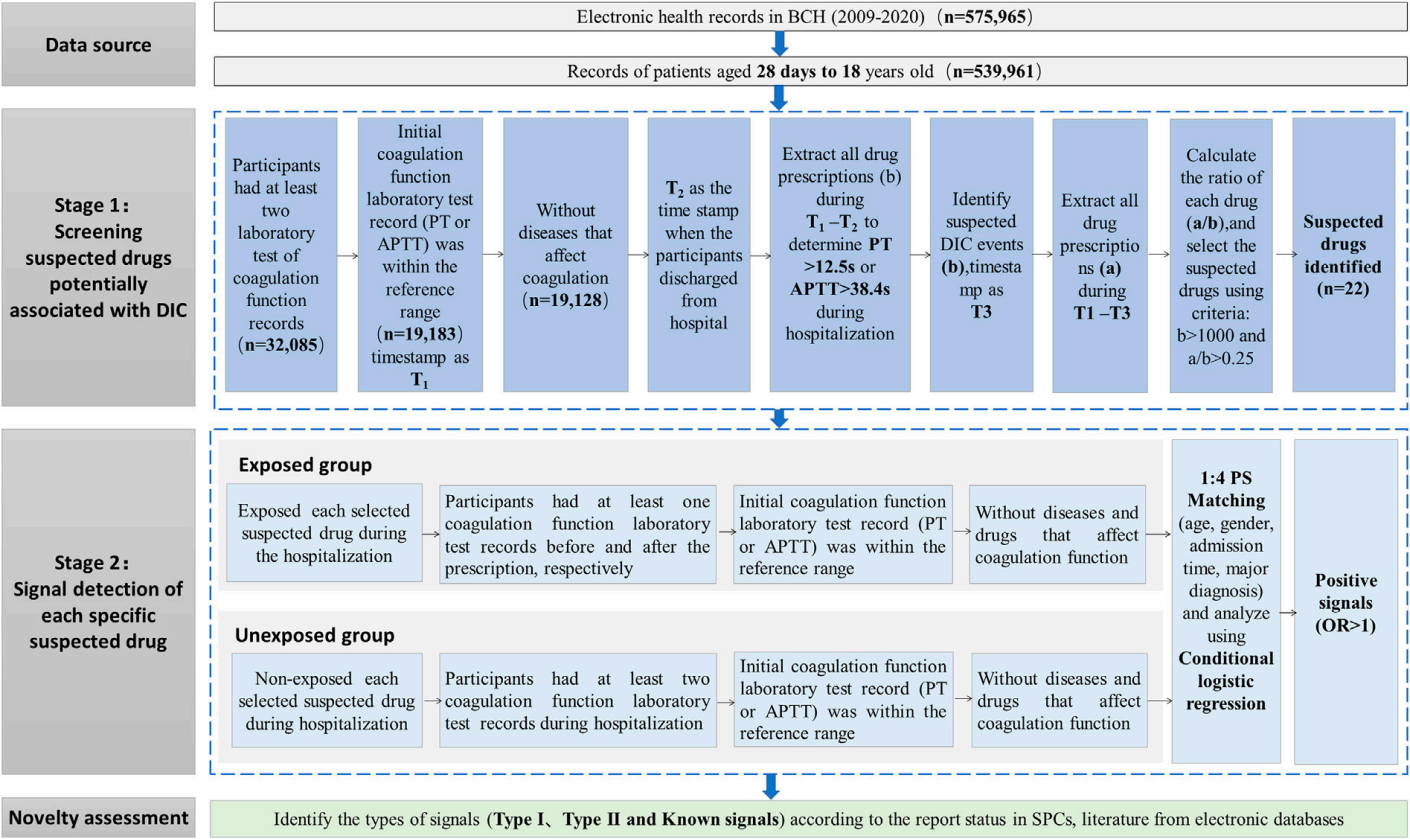
Workflow of the signal detection method of DIC using EMR data. Abbreviations: APTT, activated partial thromboplastin time; DIC, drug-induced coagulopathy; EMR, electronic medical records; PT, prothrombin time.

The overall workflow of this study was shown in Figure 1. All the involved drugs were unified with generic names and mapped with Anatomical Therapeutic Chemical (ATC) code. If a patient was prescribed more than two drugs in one record, we counted the number of users for each drug, respectively. Duplicate prescriptions of the same drug in each admission were counted only once.

#### 2.4.1 Stage 1: Screening Suspected Drugs Potentially Associated With DIC

To identify suspected drugs potentially associated with DIC for further association analysis, we developed a workflow containing three main steps (see Stage 1 part in Figure 1). The main steps were as follows:

##### 2.4.1.1 Identification of the scope for initial calculation of the number of drug users

Considering the confounding by indication, we excluded the records of patients containing a diagnosis of diseases that may affect coagulation function (shown in Supplementary Table S1). The remaining hospitalization records were defined as Group 1. The time when a patient in Group 1 obtained an initial normal result of PT or APTT after admission to the hospital was signed as timestamp 1 (T_1_), and the time for discharge of each hospitalization of every involved patient was labeled as timestamp 2 (T_2_). We calculated the number of drug users (b) during the period of T_1_-T_2_. Considering the temporal relationship between drugs and adverse reactions, we calculated the number of each kind of medication separately if a patient administered some kinds of medications at one hospitalization record before the pre-defined trigger of coagulopathy occurred.

##### 2.4.1.2 Detection of the number of potential DIC events

The hospitalization records of patients in Group 1, which were potential DIC events during T_1_-T_2_ according to the definition of DIC trigger, were included in Group 2. We labeled the time of PT longer than 12.5 s or APTT longer than 38.4 s as timestamp 3 (T_3_). Then, the number of users for each medicine in Group 2 who were identified by the DIC trigger (a) during the period of T_1_-T_3_ was calculated.

##### 2.4.1.3 Calculation of the crude ratio of potential DIC events

The ratio a/b for each drug was calculated. The suspected drug met the following criteria and was selected for further association analysis: (Peralta et al., 2019) set the threshold of a/b ratio according to the range of a/b values of solvents for intravenous infusions, such as normal saline and glucose injection, which can be regarded as the value of background since it is well known that normal saline and glucose injection have no effect on DIC; (Nalezinski, 2022) number of total users (b) >1,000, ensuring sufficient sample size and adequate power. Considering the a/b values of solvents for intravenous infusions, such as normal saline and glucose injection, ranged from 0.125 to 0.243, which can be regarded as the value of background since it is well known that normal saline and glucose injection have no effect on DIC, we set the screening threshold value of a/b ratio for suspected drugs as 0.250.

#### 2.4.2 Stage 2: Signal Detection of Suspected Drug

According to the aforementioned screening procedure of suspected drugs, we conducted a series of retrospective propensity score-matched cohort studies to detect the association between suspected drugs and DIC by comparing differences in coagulopathy event rates between the exposed and unexposed groups. Each suspected drug detected from Stage 1 was considered as an exposure and was examined in a cohort study according to the following procedures. The overall main framework is displayed in Stage 2 part in Figure 1.

##### 2.4.2.1 Algorithm defined exposed group

The eligible participants were required to be prescribed a specific screened drug after admission to BCH and had at least one PT or APTT result before and after taking the specific suspected drug, respectively. The date of initial prescription of a specific drug was considered the index time for the corresponding participant, and eligible participants should have an initial PT result within 9.4–12.5 s or an initial APTT result within 25.1–38.4 s before the index time. To accurately assess the drug-coagulopathy associations, patients who were diagnosed with diseases that may affect coagulopathy function (shown in Supplementary Table S1) or received prescriptions of anticoagulation (shown in Supplementary Table S2) (Hiensch and Lee, 2021) before the first abnormal test of coagulation index PT or APTT were also excluded.

##### 2.4.2.2 Algorithm defined unexposed group

The patients without prescriptions of specific suspected drugs were initially selected for the unexposed group. Among them, we chose the participants with at least two records of laboratory results of PT or APTT tests from admission to discharge and had an initial result of PT or APTT within the reference interval (PT: 9.4–12.5 s; APTT: 25.1–38.4 s). For the same selection considerations as the exposure group, we excluded patients diagnosed with potential coagulopathy diseases or who had prescriptions of agents which affect the coagulation function.

##### 2.4.2.3 Follow-up of the cohort

Follow-up of each cohort ended until the first occurrence of the following events: the library index of PT > 12.5 s or APTT > 38.4 s after administration, discharged from hospital, or until 31 December 2020.

##### 2.4.2.4 Propensity score matching

Given that some important variables (such as age, gender, and underlying diseases) may be imbalanced between two compared groups of this observational study, the propensity score matching method was conducted to balance the baseline characteristics of each screened suspected drug group and the unexposed group. We calculated propensity scores for the initial prescription of a specific suspected drug using the logistic regressions. The variables included in the model included age, gender, admission time, and major diagnosis (based on the classification in ICD-10). Patients with missing values for age, gender, and admission date were excluded from the analysis. For a particular suspected drug, the records from the exposed group were matched 1:4 to those of the unexposed group using the caliper matching method (caliper equaled 0.1).

##### 2.4.2.5 Signal detection

We compared the OR of DIC in each specific suspected drug cohort with the corresponding unexposed group cohorts using conditional logistic regression models. The odds ratio (OR) and its 95% confidence interval (CI) were estimated to assess the association between specific suspected drugs and the incidence of coagulopathy events. The signal of DIC was positive if the lower limit of the 95% CI of OR was greater than 1.0; otherwise, it was regarded as a negative signal.

### 2.5 Signal Novelty Assessment

One of the important steps in assessing adverse drug reactions is the evaluation of biological mechanisms. Since there was no recognized gold standard for evaluating the relevance of the DIC association, we performed a manual review of the summary of product characteristics (SPCs) included in the Drugs@FDA: FDA-approved drugs, Micromedex, the specification of drugs in China (https://www.yaozh.com/), and electronic literature databases, including PubMed, Embase, and China National Knowledge Infrastructure and Wanfang Database. We applied a combination of keywords and mesh words of generic names for each positive signal drug and adverse event, such as “coagulopathy,” “coagulation defects,” “coagulation disorders,” “coagulation dysfunction,” “hypocoagulability,” and “hypoprothrombinemia.” According to the report status in SPCs and literature from electronic databases, we defined two types of new DIC signals for children: 1) the specific drug DIC signal had never been reported in the summary of product characteristics or in the literature; 2) the specific drug signal had been reported in the literature about adults, but no reports about children could be found in the literature.

### 2.6 Statistical Analysis

The primary association analysis was conducted by conditional logistic regression and the propensity score matching method. To evaluate the robustness of the primary results, we also performed sensitivity analyses using unconditional logistic regression and the propensity score regression method other than matching in the primary analysis.

All *p* values were 2-sided, and *p* < 0.05 was considered significant for all tests. MySQL software version 14.14 (Oracle, California, United States) was used as the database management system to extract the required data from BCH’s EMR database. Data were processed and summarized using the pandas v1.2.2 model in Python 3.7. R 3.5.2 software (R Foundation for Statistical Computing, Vienna, Austria. ISBN 3-900051-00-3) was used for statistical analysis, and SAS 9.4 TS Level M5 (SAS Institute Inc., Cary, NC, United States) was used for the forest plot demonstrating the results of association analysis.

## 3 Results

### 3.1 Selection of Suspected Drugs

After combining drugs with the same ingredients as ATC but different dosages and forms, 340 drugs remained. Among these drugs, 101 satisfied the screening criteria that the total number of drug users was >1,000. Then, the a/b crude ratio was calculated. Considering the a/b values of solvents for intravenous infusions, such as normal saline and glucose injection, ranged from 0.125 to 0.243, which can be regarded as the value of background since it is well known that normal saline and glucose injection have no effect on DIC, we set the screening threshold value of a/b ratio as 0.250. Twenty-two drugs were considered suspected drugs and chosen for DIC signal detection in Stage 2. These were acetaminophen, meropenem, phenobarbital, cefoperazone sodium sulbactam sodium, fluconazole, voriconazole, ambroxol hydrochloride, salbutamol sulfate, vancomycin, ribavirin, furosemide, iodixanol, nifedipine, chlorpheniramine, cefamandole, ibuprofen, ceftizoxime, omeprazole, ceftriaxone, cetirizine, latamoxef sodium, and sulfamethoxazole. The selected suspected drugs are shown in Table 1.

**TABLE 1:** Suspected drugs associated with coagulopathy in the pediatric population.

Drug name	Pharmacological classification	ATC code	Number of DIC events (a)	Total number of usages (b)	Ratio (a/b)
Acetaminophen	Antipyretic	N02BE01	750	1,569	0.478
Meropenem	Beta-lactam antibiotic	J01DH02	1,090	2,366	0.461
Phenobarbital	Sedative-hypnotic	N03AA02	535	1,196	0.447
Cefoperazone sulbactam	Beta-lactam antibiotic	J01DD12	1,382	3,098	0.446
Fluconazole	Antifungal drug	J02AC01	1,002	2,335	0.429
Voriconazole	Antifungal drug	J02AC03	772	1,800	0.429
Ambroxol hydrochloride	Expectorant	R05CB06	2,532	5,940	0.426
Salbutamol sulfate	Asthmatic	R03AC02	1,220	3,090	0.395
Vancomycin	Polypeptide antibiotic	J01XA01	1,781	4,599	0.387
Ribavirin	Antiviral agent	J05AP01	429	1,150	0.373
Furosemide	Diuretic	C03CA01	3,345	9,161	0.365
Iodixanol	Contrast agent	V08AB09	381	1,072	0.355
Nifedipine	Calcium channel blocker	C08CA05	422	1,205	0.350
Chlorpheniramine	Antihistaminic	R06AB04	1,419	4,160	0.341
Cefamandole	Cephalosporin	J01DC03	2,355	7,066	0.333
Ibuprofen	Antipyretic	M01AE01	2,832	8,524	0.332
Ceftizoxime	Cephalosporin	J01DD07	496	1,511	0.328
Omeprazole	Mucosal protective agent	A02BC01	1,206	3,704	0.326
Ceftriaxone	Cephalosporin	J01DD04	529	1,700	0.311
Cetirizine	Antihistaminic	R06AE07	508	1,664	0.305
Latamoxef sodium	Beta-lactam antibiotic	J01DD06	1,851	6,192	0.299
Sulfamethoxazole	Sulfonamides and trimethoprim	J01EE01	1,010	3,917	0.258

DIC, drug-induced coagulopathy; ATC, anatomical therapeutic chemical.

### 3.2 Association of Suspected Drugs and Coagulopathy

The results of data extraction for the suspected drugs for each step are presented in Supplementary Table S3. For detection of the DIC signals, the median number of patients enrolled in the drug exposure groups was 492 [interquartile range (IQR): 345–826] ranging from 183 (phenobarbital) to 2,182 (furosemide), and the median number of patients enrolled in the comparison groups was 6,646 (IQR: 5,923–7,155) ranging from 4,394 (furosemide) to 7,294 (phenobarbital). The basic clinical information between two groups of each drug before and after PS matching is given in Supplementary Table S4, respectively.

**FIGURE 2:**
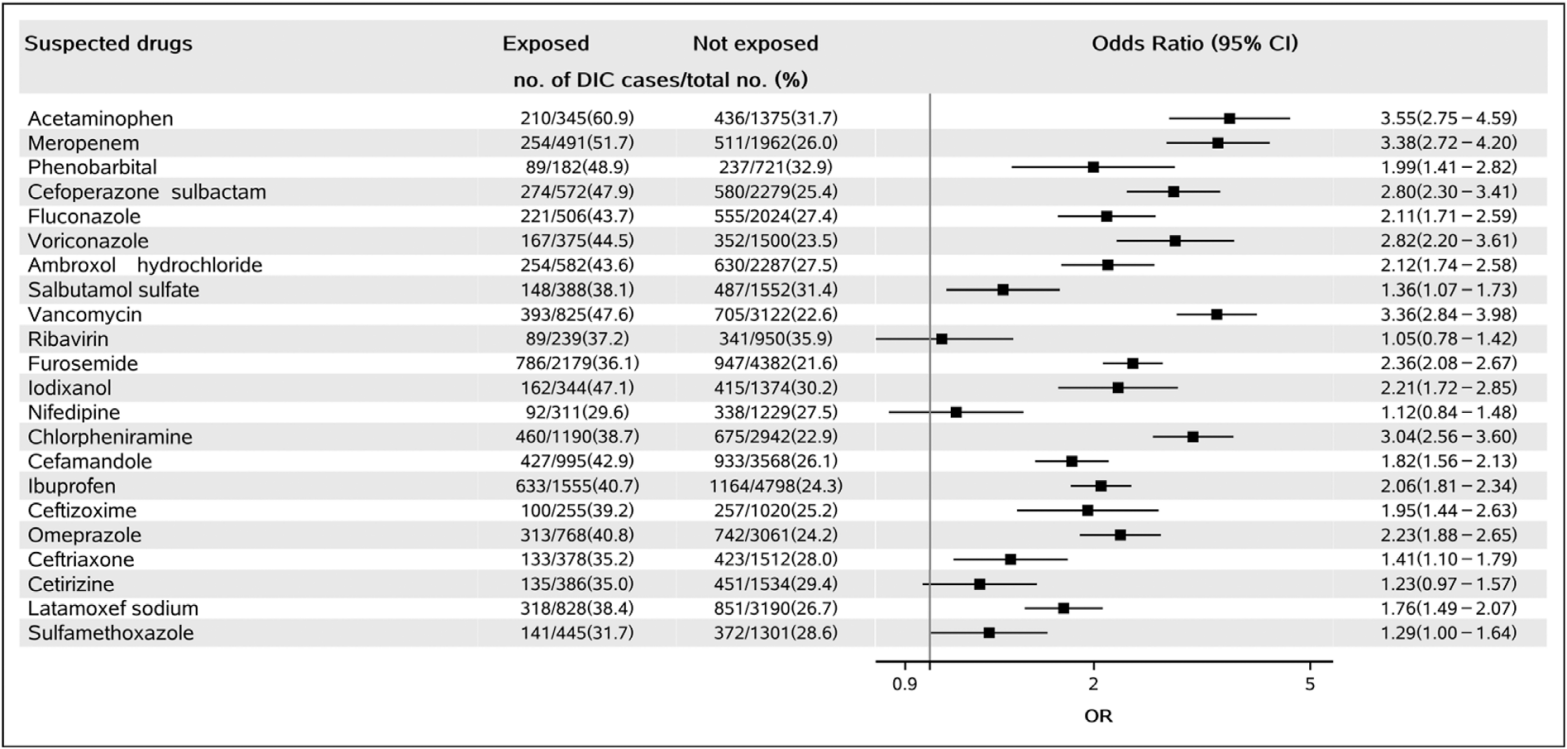
Forest plot for the association of suspected drugs and DIC. Abbreviations: DIC, drug-induced coagulopathy.

Of the 22 suspected drugs, 19 showed a positive signal, including 10 anti-infective drugs (meropenem, cefoperazone sulbactam, fluconazole, voriconazole, vancomycin, cefamandole, ceftizoxime, ceftriaxone, latamoxef sodium, and sulfamethoxazole, all OR > 1.00, *p* < 0.001, see details in Figure 2), two antipyretics (acetaminophen, OR: 3.55, 95% CI: 2.75–4.59, *p* < 0.001; ibuprofen, OR: 2.06, 95% CI: 1.81–2.34, *p* < 0.001), one sedative-hypnotic (phenobarbital, OR: 1.99, 95% CI: 1.41–2.82, *p* < 0.001), one expectorant (ambroxol hydrochloride, OR: 2.12, 95% CI: 1.74–2.58, *p* < 0.001), one asthmatics (salbutamol sulfate, OR: 1.36, 95% CI: 1.07–1.73, *p* < 0.001), one diuretics (furosemide, OR: 2.36, 95% CI: 2.08–2.67, *p* < 0.001), one contrast agent (iodixanol, OR: 2.21, 95% CI: 1.72–2.85, *p* < 0.001), one antihistaminic (chlorpheniramine, OR: 3.04, 95% CI: 2.56–3.60, *p* < 0.001), and one mucosal protective agent (omeprazole, OR: 2.23, 95% CI: 1.88–2.65, *p* < 0.001). The remaining three drugs (ribavirin, nifedipine, and cetirizine) were found to be not associated with DIC. The detailed results of all the 22 drugs-DIC associations are shown in Figure 2.

Results from sensitivity analyses also showed the same results for each drug with the primary analysis conducted by the propensity score matching method. Nineteen drugs were potentially associated with DIC, and three drugs were not statistically associated with DIC (Supplementary Table S5).

### 3.3 Signal Novelty Evaluation

The novelty of 19 positive DIC signals observed in Stage 2 was further evaluated through SPCs and current literature (Table 2). Three drugs, namely salbutamol sulfate, chlorpheniramine, and omeprazole, were found to be new DIC type I signals, as the coagulopathy event had never been reported in the literature, neither in children nor adults. In addition, twelve drugs, namely meropenem, cefoperazone sulbactam, fluconazole, voriconazole, ambroxol hydrochloride, furosemide, iodixanol, cefamandole, ceftizoxime, ceftriaxone, latamoxef sodium, and sulfamethoxazole, were considered new signals for type II DIC. Coagulation disorders associated with these 12 drugs have not been found in pediatric patients but have been reported in adults. The remaining four drugs have been reported to be associated with coagulation dysfunction in both adult and pediatric patients.

**TABLE 2:** The novelty of the positive signals of DIC.

Suspected drugs	Literature of PubMed/Embase^a^		Literature of Chinese database (CNKI/Wangfang)^a^		SPCs^b^	Signal type^c^
	Adults	Children	Adults	Children		
Acetaminophen	**√**	**√**	**√**	**×**	**√**	Known
Meropenem	**√**	**×**	**√**	**×**	**×**	II
Phenobarbital	**√**	**√**	**×**	**×**	**×**	Known
Cefoperazone sulbactam	**√**	**×**	**√**	**×**	**√**	II
Fluconazole	**√**	**×**	**√**	**×**	**√**	II
Voriconazole	**√**	**×**	**√**	**×**	**×**	II
Ambroxol hydrochloride	**×**	**×**	**√**	**×**	**×**	II
Salbutamol sulfate	**×**	**×**	**×**	**×**	**×**	I
Vancomycin	**√**	**×**	**×**	**√**	**×**	Known
Furosemide	√	**×**	**×**	**×**	**×**	II
Iodixanol	**√**	**×**	**√**	**×**	**×**	II
Chlorpheniramine	**×**	**×**	**×**	**×**	**√**	I
Cefamandole	**√**	**×**	**√**	**×**	**×**	II
Ibuprofen	**√**	√	√	√	**√**	Known
Ceftizoxime	**√**	**×**	**×**	**×**	**×**	II
Omeprazole	**×**	**×**	**×**	**×**	**×**	I
Ceftriaxone	**√**	**×**	**√**	**×**	**×**	II
Latamoxef sodium	**√**	**×**	**√**	**×**	**×**	II
Sulfamethoxazole	**√**	**×**	**×**	**×**	**×**	II

DIC, drug-induced coagulopathy; SPCs, summary of product characteristics.

a Literature reviewed: 1) PUBMED: https://pubmed.ncbi.nlm.nih.gov; 2) Embase: https://www.embase.com; 3) Wanfang: https://www.wanfangdata.com.cn/index.html; 4) CNKI: https://www.cnki.net.

b SPCs reviewed: 1) Micromedex: https://www.ibm.com/watson-health/learn/micromedex; 2) FDA website: https://www.fda.gov/; (3) Drug instructions: https://www.yaozh.com/.

c Signal type I: The specific drug-DIC signal had never been reported in the summary of product characteristics or in the literature; II: the specific DIC signal had been reported in the literature about adults, but no reports about children could be found in the literature; known: the specific drug-DIC association had been reported.

## 4 Discussion

Early recognition of the cause of coagulation disorder is critical to making appropriate treatment and saving patients’ lives. Drug-induced coagulopathy is a type of acquired coagulopathy that has been associated with some medications and can lead to devastating consequences for the patient, especially for critically ill patients (Levi and Schultz, 2010). Often, the cause of DIC is not recognized in a timely manner, resulting in recurrent coagulation disorders and inappropriate treatments. Drug-induced coagulopathy and bleeding have similar principles of management including protocols calling for early diagnosis and timely reversal of coagulopathy with antidotes (Nalezinski, 2022). Greater publicity will increase awareness and suspicion of DIC among pediatricians and improve clinicians’ ability to evaluate, accurately diagnose, and manage patients who present with unexpected coagulopathy (Nalezinski, 2022) because a delay in recognition can lead to significant morbidity and mortality; clinical criteria such as the Naranjo Adverse Drug Reaction Probability Scale were used to help determine the risk of DIC, which was less efficient. By contrast, our algorithm based on EMR data could be a referential experience to provide more clues for pediatric drug post-marketing pharmacovigilance. We found nineteen positive signals of DIC, including twelve new signals in a pediatric population. Considering the precision medicine in pediatrics, when children are treated with such drugs, health professionals should be aware of the potential coagulation disorder risk and monitor coagulation parameters during clinical therapy with these suspected drugs, particularly the need to monitor AT and APTT. In addition, these drugs may be the suspected drugs for post-marketing surveillance and regulation, which could be the candidate target drugs for further signal validation studies.

### 4.1 New Signals of DIC

Using our established two-stage algorithm, the association of omeprazole, salbutamol sulfate, and chlorpheniramine with DIC was found to be a potential three new type I positive signals in this study for the first time. Although there have been no published reports about these potential drug-coagulopathy event pairs neither in pediatrics nor in adults, some other clues could indicate these potential associations might exist. FDA has initiated two phase IV clinical studies about omeprazole and coagulopathy, one is focused on coagulopathy and drugs of ingredients of omeprazole, and another is about omeprazole and coagulopathy (eHealthMe, 2022; eHealthMe) using real-world post-marketing data. With real-world medical big data and proven AI algorithms, eHealthMe provides a platform for everyone to run phase IV clinical trials. According to the latest updated data on 21 April 2022, 4,63,527 people reported having side effects when taking drugs with ingredients of omeprazole. Among them, 850 people (0.18%) had coagulopathy. Similar post-marketing safety reports on salbutamol sulfate could be searched in the SIDER 4.1 database (Resource), which contains available information including side effect frequency, drug, and side effect classifications as well as links to further information on marketed medicines and their recorded adverse drug reactions. The information is extracted from public documents and package inserts. Considering the new signal of chlorpheniramine, the adverse effects listed in its SPCs include drowsiness, thirst, polyuria, sore throat, drowsiness, weakness, palpitations, ecchymosis of the skin, and bleeding tendency. A rare case report suggested that acquired hemophilia due to factor VIII inhibitor(s) should be considered in the appropriate setting when patients present with unexplained and even minor bleeding while on treatment with acetaminophen or chlorpheniramine alone or combined (Famularo et al., 2004). Since this rare case was unclear whether exposure to those compounds triggered an autoimmune response against factor VIII although there has been no clinical or laboratory evidence in this patient of liver dysfunction, which ruled out the hypothesis of factor VIII coagulopathy caused by acetaminophen-induced impairment of liver metabolism. The use of the Naranjo Probability Scale indicated a possible relationship between acquired hemophilia and exposure to acetaminophen and chlorpheniramine therapy in this patient. Further investigations about the potential association between chlorpheniramine and coagulopathy are still needed. It should be noted that general ADR was defined as “an appreciably harmful or unpleasant reaction, resulting from an intervention related to the use of a medicinal product” (Edwards and Aronson, 2000), which includes drug side effects and toxic effects (toxicity), allergic reactions, diathesis, double infection induced by anti-infection drugs, dependency, and carcinogenic, teratogenic and mutagenic effects, etc. Considering overdose drug use is more common in children’s clinical practice, further mechanism search is urgent to validate type I DIC signals detected in our studies.

Other twelve drug-DIC associations (meropenem, cefoperazone sulbactam, fluconazole, voriconazole, ambroxol hydrochloride, furosemide, iodixanol, cefamandole, ceftizoxime, ceftriaxone, latamoxef sodium, and sulfamethoxazole) were identified as potentially new type II signals in children. Among them, cefamandole, ceftizoxime, and ceftriaxone are cephalosporins, while cefoperazone sulbactam is a compound preparation of cephalosporin. Latamoxef sodium belongs to the β-Lactam family of antibiotics, and its antibacterial spectrum and antibacterial action are like that of the third-generation cephalosporin. Cephalosporins that contain the N-methylthiotetrazole side chain (NMTT-cephalosporin) have been reported to be associated with coagulation-related adverse events, especially hypoprothrombinemia or PT prolongation in patients with underlying clinical conditions at risk for bleeding (Park et al., 2019). Due to the chemical structure of the NMTT, this side chain interferes with vitamin K metabolism and results in a decrease in prothrombin synthesis and a corresponding decrease in thrombin synthesis. Hypoprothrombinemia is characterized by a deficiency of the clotting factor prothrombin and presents an elevated PT level and a prolonged APTT (Agnelli et al., 1986). Some adult case reports have documented cephalosporin-induced acute coagulopathy (Chang, 1983; Haubenstock et al., 1983; Cuxart et al., 1987; Nichols et al., 1987; Li et al., 2017; Hu, 2019; Wang et al., 2020; Li et al., 2021) but few about the pediatric population. The incidence of vitamin K-dependent coagulopathy associated with NMTT-containing antibiotics was shown to range from 2.2 to 19% while the incidence of bleeding associated with non-NMTT-containing antibiotics such as sulfamethoxazole (SMX) and meropenem was shown to range from 0 to 4% (Shevchuk and Conly, 1990; Cook and Ponte, 1994; Fotouhie et al., 2016; Furumi et al., 2019; Guo et al., 2022). Therefore, treatment with antibiotics, including non-NMTT-containing antibiotics and cephalosporins, should be remembered as potential causes of vitamin K deficiency. The liver is the main synthesis site of most coagulation factors (factor II, V, VII, IX, XII, fibrinogen, and fibrinolytic progenitor) and inhibitory proteins (α2 anti-fibrinolytic enzyme, antithrombin, protein C, protein S, etc.). Oral azole antifungal medications including fluconazole and voriconazole could affect liver function to reduce vitamin K absorption and result in deficiency of vitamin K-dependent factors II, VII, X, and IX (Lo Re et al., 2016). There had been some adults case reports about the coagulopathy events induced by ambroxol hydrochloride (Hu et al., 2003), furosemide (Hilden and Amris, 1968; Averina and Alieva, 1972), and iodixanol (Bai et al., 2021), but few of them tried to explain the mechanism of drug adverse reaction. Although our results were the first to show that these aforementioned twelve type II signal drugs might be associated with adverse coagulopathy in children, these findings will need further investigation to be confirmed and explained.

### 4.2 Strength and Limitation

Strengths of this study are its large size and longitudinal integrated multi-source data from the hospital information systems, biochemical laboratory, and drug prescription records with detailed clinical data. The inadequacy of the spontaneous reporting system (SRS) has led many countries to implement pharmacovigilance systems to enhance their risk management capacity. Compared with the proposed tool with those based on the SRS, the active surveillance based on the routinely collected data integration is an effective approach for pharmacovigilance, which can detect previously unrecognized adverse drug signals in the real practice immediately, as well as provide more detailed information about symptoms, signs, diagnosis, timing sequence, and medication to analyze the potential association for drug-ADR pairs. Also of note, Yoon et al. (2012) established a series of electronic health record (EHR)-based pharmacovigilance methods called the BASE, CLEAR, and MetaLAB for laboratory abnormalities (Lee et al., 2017). Our study used a two-stage data-driven drug screening and PS matching method to detect children’s DIC signals. It is important to realize that this is a tool to assist with detection but does not ensure the identification of ADRs. In comparison with the CLEAR method, our 2-stage designed approach has several advantages. In the process of selecting the drugs suspected to cause DIC, we assessed the potentialities by computing the crude incidence of ADEs in drug users. This crucial additional step increased the efficiency and speed of subsequent steps. In addition, more complicated confounders, such as relevant diagnoses with clear competing causes and medications that may affect the level of relevant laboratory indicators and induce confounding by indication, were excluded to enhance the reliability and accuracy of the results. These results suggested that our method is a valuable tool to facilitate earlier signal detection using routinely collected EMR data.

This study also had some limitations inherent to hospital-based retrospective observational data; hence, there is always a chance of unmeasured residual confounding. The signal detection using our method was dependent on the time for measurement of PT or APTT, and the sequence relationship between these signals and the actual occurrence of DIC may be inverted due to the possible delayed detection. Dose-related effects and possible residual confounders, such as those not controlled, lead to potential bias. Third, since our study is only based on EMR data from a single-center and coagulation tests are not routine laboratory tests, the sample size of exposure to both specific drugs and PT/APTT tests, such as phenobarbital, was small and limited, which could lead to poor representation of results. Therefore, the external data validation and distributed signal detection method based on multi-center EMR data should be further discussed in the future.

After two decades of implementation of the drug-related adverse reaction reporting system, China has formally implemented a pharmacovigilance system with the pharmacovigilance quality management standards and a series of supporting technical documents created to improve the safety of medication given to patients (Song et al., 2022). Since the development of children’s medicine cannot meet clinical needs, potential off-label drug use may occur in children, and this risk should not be ignored (Moore-Hepburn and Rieder, 2021). Access to high-quality data from multiple sources will lead to more comprehensive and scientific risk assessment and the generation of reliable scientific evidence for drug safety regulatory decisions. China has improved its spontaneous reporting system by installing the China Hospital Pharmacovigilance System (CHPS) module in hospital information systems (HIS) of some sentinel tertiary hospitals. The main function of the CHPS module is to automatically capture most of the information needed for the ADR report form from the hospital’s HIS system, which will lead to significant time savings for HCPs completing ADR reports and reduce the omission of important information during the filing process. On the other hand, bringing together a wealth of real-world data to build new pharmacovigilance systems has proven the possibility of successfully detecting and assessing safety signals and effectively enhancing pharmacovigilance capabilities. At present, we have developed an automated program based on this algorithm. Furthermore, in the next step, more attention will be paid to integrating these multiple modules into a drug safety monitoring platform to support quick-response tools for pediatric clinicians and pharmacists in multi-center hospitals through a common data model (CDM), just like the Sentinel Initiative of FDA. Future research will also focus on tighter integration of the structured data and clinical narratives in EMR data to improve the accuracy and scalability of the method.

## 5 Conclusion

In this study, we developed a two-stage designed pharmacovigilance method to explore potentially DIC signals using routine EMR data. Fifteen positive signals of DIC, including twelve new signals in children, were detected. Our work promotes the application of EMR datasets in pharmacovigilance and precision medicine in pediatrics.

## Data Availability

The original contributions presented in the study are included in the article/Supplementary Material; further inquiries can be directed to the corresponding authors.
