# Supplementary material for: Signal Detection of Pediatric Drug–Induced Coagulopathy Using Routine Electronic Health Records
Source: Front Pharmacol. 2022 Jul 20;13:935627. doi: 10.3389/fphar.2022.935627 (PMC9348591; doi:10.3389/fphar.2022.935627)
Supplement: Supplementary file 1 [file DataSheet1.docx]

**Table S1. The excluded diseases with clear competing effects of coagulation [1]**

| **Category** | **ICD-10 Codes** |
| --- | --- |
| Aplastic anemia | D61.905、D61.903、D61.801 、D61.101、D61.003、D61.001、D60.901、D59.501 |
| Autoimmune hemolytic anemia | D59.102、 D59.051、D59.251 |
| Autoimmune hepatitis | K75.809 |
| Bone marrow proliferation | C94.451、C94.051 |
| Chronic liver disease | E14.652、K71.051、K71.351、K71.552、K71.851、K76.901、 E05.903+ |
| Disseminated intravascular coagulation | D65.X01、P60.X01 |
| Hemolytic uremic syndrome | D59.301 |
| Hypersplenism | R94.853 |
| Langerhans cell histiocytosis | D76.304 |
| Leucocythemia | C90.1、C91.0、C91.1、C91.2、C91.4、C92.-、C93.0、C93.1、C93.3、C93.7、C93.9、C94.2、C94.3 |
| Liver cirrhosis | K74.151、K74.251、K74.654 |
| Lymphoid hyperplasia | D82.351 |
| Lymphoma | C91.5、C96.3、C85.7、C84.5、C84.4、C84.3、C84.2、C82.7 |
| Myeloproliferative disorder | D46.901、D46.751 |
| Myelosuppression | D75.808、 D75.805、D61.902、D61.901 |
| Rheumatoid arthritis | M05.301、M05.302、 M05.306+、M05.951、M06.001、M06.991、M08.001 |
| Scleroderma | L94.052、L94.151、M34.005、M34.801+、M34.901、M34.902 |
| Systemic lupus erythematosus | M32.006、M32.155+、M32.901、P00.852 |
| Vasculitis | I77.502、I77.605、I77.607、M31.003、D69.001 |

Abbreviations: ICD-10, international classification of diseases (Version 10).

**Table S2. The excluded drugs with effects of coagulation [1]**

| **ATC** | **Drugs** | **Dosage form** | |
| --- | --- | --- | --- |
| B02AA03 | Aminomethylbenzoic acid | | Injection |
| B02BD05 | Recombinant human coagulation factor VII | | Injection |
| B02BD02 | Human coagulation factor VIII | | Injection |
| B02BD06 | von Willebrand factor and coagulation factor VIII in combination | | Injection |
| B02BD01 | Coagulation factor IX, II, VII and X in combination | | Injection |
| B02BD01 | Coagulation factor VIIa | | Injection |
| - | Human prothrombin complex | | Injection |
| B01AD04 | Urokinase | | Injection |
| B05AX02 | Streptokinase | | Injection |

# Table S3. The data filtering workflow for suspected drugs

| **Drug name** | **Exposed to suspected drug** | **At least 1 coagulation function test before and after medication** | **Initial PT /APTT within ULN** | **Exclude diseases that affect coagulation** | **Exclude drugs that affect clotting function** | **Not exposed to suspected drug** | **At least 2  PT /APTT tests** | **Initial PT /APTT tests within ULN** | **Exclude diseases that affect** **coagulation** | **Exclude drugs that affect clotting function** |
| --- | --- | --- | --- | --- | --- | --- | --- | --- | --- | --- |
| Acetaminophen | 12593 | 2275 | 417 | 355 | 345 | 527368 | 29114 | 8111 | 7321 | 7184 |
| Meropenem | 14555 | 2715 | 707 | 514 | 492 | 525406 | 28158 | 7613 | 7013 | 6886 |
| Phenobarbital | 6142 | 1024 | 219 | 197 | 183 | 533819 | 30518 | 8258 | 7427 | 7294 |
| Cefoperazone sulbactam | 28166 | 3104 | 733 | 597 | 572 | 511795 | 26821 | 7392 | 6723 | 6604 |
| Fluconazole | 11029 | 2411 | 638 | 525 | 507 | 528932 | 28571 | 7788 | 7062 | 6929 |
| Voriconazole | 6695 | 1505 | 539 | 388 | 375 | 533266 | 30145 | 7953 | 7284 | 7148 |
| Ambroxol hydrochloride | 68013 | 4515 | 729 | 625 | 587 | 471948 | 22823 | 6730 | 6017 | 5923 |
| Salbutamol sulfate | 30305 | 2172 | 476 | 414 | 390 | 509656 | 28196 | 7553 | 6765 | 6646 |
| Vancomycin | 19883 | 3338 | 1142 | 846 | 826 | 520078 | 27347 | 7133 | 6647 | 6521 |
| Ribavirin | 11960 | 1145 | 292 | 246 | 241 | 528001 | 30038 | 8224 | 7410 | 7267 |
| Furosemide | 75094 | 10308 | 2700 | 2225 | 2182 | 464867 | 19595 | 4695 | 4431 | 4394 |
| Iodixanol | 10915 | 1203 | 383 | 347 | 344 | 529046 | 30452 | 8136 | 7325 | 7185 |
| Nifedipine | 6138 | 1420 | 453 | 341 | 314 | 533823 | 30328 | 8013 | 7267 | 7155 |
| Chlorpheniramine | 35312 | 5278 | 1597 | 1213 | 1190 | 504649 | 25828 | 6674 | 6276 | 6153 |
| Cefamandole | 75259 | 5105 | 1209 | 1022 | 998 | 464702 | 22494 | 6245 | 5747 | 5672 |
| Ibuprofen | 89868 | 9996 | 1986 | 1606 | 1561 | 450093 | 18643 | 5309 | 4939 | 4851 |
| Ceftizoxime | 14348 | 1513 | 335 | 263 | 255 | 525613 | 29234 | 8061 | 7329 | 7193 |
| Omeprazole | 57862 | 3432 | 982 | 793 | 771 | 482099 | 26733 | 7025 | 6393 | 6287 |
| Ceftriaxone | 25419 | 1274 | 415 | 390 | 379 | 514542 | 29464 | 7820 | 7003 | 6882 |
| Cetirizine | 16184 | 1757 | 464 | 397 | 387 | 523777 | 29408 | 7957 | 7160 | 7021 |
| Latamoxef sodium | 50560 | 3353 | 1007 | 846 | 828 | 489401 | 25491 | 6589 | 6009 | 5906 |
| Sulfamethoxazole | 51402 | 2306 | 627 | 451 | 446 | 488559 | 23386 | 5359 | 5127 | 5001 |

**Abbreviations**: PT: prothrombin time; APTT: activated partial thromboplastin time; ULN: upper limit of normal.

**Table S5. Sensitivity analyses of associations between suspect drugs-and coagulopathy using PS regression**

| **Suspect drugs** | **Exposed group** | | | **Unexposed group** | | ***β*** | ***P* value** | **OR (95%CI)** |
| --- | --- | --- | --- | --- | --- | --- | --- | --- |
|  | number of DIC events | | number of non- DIC events | number of DIC events | number of non- DIC events |  |  |  |
| Acetaminophen | 210 | | 135 | 1943 | 5228 | 1.297 | <0.001 | 3.658(2.916,4.602) |
| Meropenem | 254 | | 237 | 1788 | 5084 | 1.125 | <0.001 | 3.080(2.548,3.725) |
| Phenobarbital | 89 | | 93 | 1995 | 5284 | 0.772 | <0.001 | 2.163(1.591,2.938) |
| Cefoperazone sulbactam | | 274 | 298 | 1649 | 4942 | 1.016 | <0.001 | 2.761(2.312,3.297) |
| Fluconazole | 221 | | 286 | 1830 | 5085 | 0.749 | <0.001 | 2.115(1.751,2.552) |
| Voriconazole | 167 | | 208 | 1970 | 5163 | 1.012 | <0.001 | 2.752(2.207,3.427) |
| Ambroxol hydrochloride | 256 | | 331 | 1494 | 4418 | 0.645 | <0.001 | 1.905(1.590,2.280) |
| Salbutamol sulfate | 148 | | 240 | 1789 | 4845 | 0.298 | 0.007 | 1.347(1.081,1.672) |
| Vancomycin | 393 | | 432 | 1665 | 4842 | 1.184 | <0.001 | 3.267(2.795,3.820) |
| Ribavirin | 90 | | 151 | 2058 | 5195 | 0.185 | 0.188 | 1.203(0.911,1.581) |
| Furosemide | 786 | | 1394 | 948 | 3435 | 0.856 | <0.001 | 2.355(2.090,2.653) |
| Iodixanol | 162 | | 182 | 1992 | 5178 | 0.667 | <0.001 | 1.948(1.560,2.430) |
| Nifedipine | 93 | | 221 | 2020 | 5120 | 0.150 | 0.256 | 1.161(0.893,1.499) |
| Chlorpheniramine | 460 | | 730 | 1616 | 4524 | 1.108 | <0.001 | 3.028(2.603,3.523) |
| Cefamandole | 427 | | 568 | 1456 | 4208 | 0.703 | <0.001 | 2.019(1.752,2.326) |
| Ibuprofen | 636 | | 922 | 1174 | 3668 | 0.760 | <0.001 | 2.138(1.889,2.419) |
| Ceftizoxime | 100 | | 155 | 2026 | 5152 | 0.557 | <0.001 | 1.746(1.34,2.264) |
| Omeprazole | 313 | | 457 | 1653 | 4624 | 0.852 | <0.001 | 2.345(1.995,2.756) |
| Ceftriaxone | 133 | | 245 | 1909 | 4961 | 0.447 | <0.001 | 1.564(1.246,1.955) |
| Cetirizine | 135 | | 251 | 1987 | 5020 | 0.204 | 0.071 | 1.226(0.980,1.527) |
| Latamoxef sodium | 318 | | 510 | 1602 | 4293 | 0.590 | <0.001 | 1.804(1.543,2.108) |
| Sulfamethoxazole | 142 | | 304 | 1518 | 3469 | 0.338 | 0.004 | 1.403(1.114,1.760) |

**Abbreviations**: DIC: Drug-induced coagulopathy

**Reference**

[1] Hiensch RJ, Lee A. Coagulopathy and Thrombocytopenia. Mount Sinai Expert Guides: Critical Care. 2021:561-574.
